# Enhancing growth performance, meat quality, and gut health of Jiuyuan Black chickens by using *Bacillus coagulans*-fermented bedding

**DOI:** 10.5713/ab.250646

**Published:** 2025-12-01

**Authors:** Liuting Wu, Xin Sun, Felix Kwame Amevor, Gang Shu, Xiaoling Zhao

**Affiliations:** 1 State Key Laboratory of Swine and Poultry Breeding Industry, College of Animal Science and Technology, Sichuan Agricultural University, Chengdu, China; 2 Farm Animal Genetic Resources Exploration and Innovation Key Laboratory of Sichuan Province, College of Animal Science and Technology, Sichuan Agricultural University, Chengdu, China; 3 Key Laboratory of Livestock and Poultry Multi-omics, Ministry of Agriculture and Rural Affairs, Sichuan Agricultural University, Chengdu, China; 4 Department of Basic Veterinary Medicine, Sichuan Agricultural University, Chengdu, China

**Keywords:** Antioxidant Capacity, *Bacillus coagulans*-fermented Bedding, Growth Performance, Gut Health, Jiuyuan Black Chicken, Meat Quality

## Abstract

**Objective:**

*Bacillus coagulans* is a spore-forming probiotic known for its resilience and metabolic activity, both of which are desirable in promoting gut health and oxidative balance. Nonetheless, the beneficial effects of *B. coagulans*-fermented bedding (BFB) on raising native Chinese chicken breed farming remain largely unknown. This study was conducted to evaluate BFB supplementation on growth performance, meat quality and gut health in Jiuyuan Black chicken.

**Methods:**

A total of 120 male chicks were allocated to the control (CON, using traditional litter) and BFB groups with four replicates per group containing fifteen birds. The chickens were monitored for 70 days; growth performance was evaluated on days 35 and 70, while meat quality, intestinal integrity, antioxidant capacity, and animal welfare were evaluated on day 70.

**Results:**

The results showed that, after 70 days, the chickens in the BFB group exhibited significantly higher average daily gain, lower feed conversion ratio, and increased semi-eviscerated yield and intramuscular fat content compared to the CON group (p<0.05). Breast muscle from the BFB group showed enhanced flavor and juiciness than the CON group (p<0.05). Furthermore, the histological analysis of the jejunum demonstrated increased villus height-to-crypt depth ratio, alongside upregulated expression of tight junction proteins (*Claudin*-1 and ZO-1) (p<0.05). Additionally, total antioxidant capacity, catalase, and superoxide dismutase activities were increased, with reduced malondialdehyde levels in the serum and jejunal tissue (p<0.05). Furthermore, BFB improved Jiuyuan Black chickens feather coverage (p<0.05).

**Conclusion:**

This study indicated that BFB treatment was a good source of reducing oxidative stress in broilers by improving gut health, antioxidant capacity and meat quality which may provide an essential proof for the practical application to enhance growth performance without causing welfare issues in poultry.

## Graphical Abstract

[Fig f11-ab-250646]

## INTRODUCTION

The poultry sector is a dynamic agricultural branch that makes a significant contribution to meeting the rapidly growing world population’s need for affordable, high-protein food [1]. The Jiuyuan Black chicken breed is native to Sichuan Province, China and originates from Wanyuan City (31°12’N, 108°30’E), is highly valued for its superior meat quality and flavor profile [2]. Wanyuan City belongs to the selenium-enriched area; as a result, the Jiuyuan Black chicken has higher levels of selenium than those in other native chickens when it comes to meat and eggs, rendering them more nutritious [3]. However, the sexual maturity of Jiuyuan Black chickens is relatively late (about 180 days old) [4]. In addition to that, similar other avian species this specie is also observed with high susceptibility to different intestinal diseases which may impair growth and performance leading toward substantial economic losses for the industry [5]. Traditionally, antibiotics have been used with high frequency in order to promote growth and for prophylaxis [6]. Despite the above advantages, concerns have arisen over antibiotic resistance and residual contamination in meat by using antibiotics as feed additives in poultry farms [7], prompting researchers to seek other alternatives for disease prevention and growth promotion on poultry farming.

Probiotics are living microorganisms which have shown potential as alternatives to antibiotics and can improve animal health when administered in efficacious concentrations [8]. Among them, *Bacillus coagulans*, a lactic acid-producing bacterial species has attracted more interest due to its survival in extreme acidic gastric condition thereby showcasing the beneficial aspects of *Lactobacillus* and *B. coagulans* stains [9]. Moreover, the litter quality in poultry breeding has remarkable affects on bird health and productivity [10]. The type of litter material has a major impact on the microbial status in chickens related to moisture content, ammonia emission and respiratory health [11]. In recent years, the fermented bedding technology is found to be an interesting farming technique which belongs from natural agriculture and microbial engineering that has high potential on improving feed conversion ratio (FCR), reducing disease incidence, and enhancing meat quality in poultry [12]. Fermenting bedding materials with beneficial microorganisms, such as *B. coagulans* is expected to subsequently result in decreased pathogen loads and greater microbial diversity [9,13].

With the increasing demands for quality and nutrition in poultry products amongst consumers, Jiuyuan Black chicken has been gaining more attraction on both meat-processed markets and egg market [4]. Despite its economic potential, research on the application of *B. coagulans*-fermented bedding (BFB) in poultry farming remains limited. Therefore, this study aims to investigate the impact of *B. coagulans* in fermented bed culture can improve the growth performance, intestinal health, and overall welfare of Jiuyuan black chickens. We hypothesized that this modified bedding will lead to improved growth rates, enhanced intestinal health, and superior oxidative stress-related indicators compared to traditional bedding materials.

## MATERIALS AND METHODS

### Ethical statement, animal use, and care

Trials were conducted strictly following the animal welfare guidelines of China. Animal experiments were performed following the Sichuan Agricultural University’s Guidelines for the Care and Use of Laboratory Animals and were approved by the Animal Ethics Committee (Approval No. 20240237; Chengdu, Sichuan, China). All immunization procedures were completed prior to trial initiation and strict biosecurity protocols and disinfection were maintained throughout the experimental period. The room temperature was maintained at approximately 23.5°C (range 22.2°C–24.3°C). Chickens were housed under a 16 h light / 8 h dark cycle with ad libitum access to feed and water.

### Experimental design

Healthy male Jiuyuan black chickens (n = 120; 80 days old), with a body weight of 1.35±0.04 kg, were randomly allocated into two groups of 60 birds each, with four replicates of 15 chickens each. All experimental groups were housed in the same room. The 10-week trial comprised two experimental groups, namely conventional bedding (CON; 50% rice husk+ 50% sawdust) and BFB (conventional bedding+*B. coagulans*) groups. The basal diet was formulated according to Chinese Feeding Standard of Chicken recommendations (Ministry of Agriculture PRC) (Supplement 1).

### Preparation of fermented bedding

Dry sawdust and clean grain hulls were mixed in a 1:1 volume ratio. Subsequently, 0.2 kg of 2.5×10^10^ CFU/kg powdered *B. coagulans* NZRJ360 (Shanghai Jiaguan Biotechnology) was added per 1 m^3^ of the bedding, and cornstarch was added at a ratio of 1:10. These materials were mixed with water for dilution and set aside. For every 1 m^2^ of floor, 0.5 m^3^ of mixed bedding was placed. After 48 h of material warming, the pile was turned to promote complete fermentation. A characteristic aroma developed after 4 to 5 days. Fermentation continued for 7 to 10 days and was considered complete when hot gas was released from the bed or when the internal temperature exceeded 50°C. Throughout the experimental period, the bedding was turned every three days, maintaining a thickness of 20 cm. The fermented bedding was prepared and set in place three days before the poultry were introduced. On day 35, *B. coagulans* was supplemented into the fermented bedding.

### Recording and sample collection

The experimental period lasted 10 weeks. Fasting body weight measurements were conducted on days 35 and 70 at 08:00. Feed was withdrawn at 20:00 the night prior to weighing, while water remained available ad libitum. At the end of the experiment, two Jiuyuan black chickens were selected from four replicates in each experimental group, weighed, and euthanized via cervical dislocation (n = 8). Blood samples were collected (5 mL) via a wing vein and centrifuged at 2,000×g for 10 min at 4°C to separate the serum. After centrifugation, the serum was collected in a 1.5-mL aseptic centrifuge tube. Following a single rinse with normal saline, the middle portion of the jejunum, approximately 2.5 cm long, was wrapped in sterile tin foil. Serum and jejunum samples were stored at −80°C for further analysis.

### Growth performance determination

On days 35 and 70, the chickens were weighed at 8:00 am. Total feed consumption for each chicken was also recorded. Feed consumption was measured per replicate and used to calculate the average daily gain (ADG), average daily feed intake (ADFI), and FCR of the Jiuyuan black chickens. Furthermore, the weights of the internal organs, including the heart, liver, spleen, kidneys, muscular stomach, and glandular stomach, and the length of the small intestine were measured. The organ index (g/kg) was calculated as follows: organ weight/body weight.

### Slaughter performance determination

On day 150, two Jiuyuan black chickens were randomly selected from each replicate (n = 8), weighed, and euthanized through exsanguination via the carotid artery following venous blood collection; samples were obtained after slaughtering. The following parameters were calculated: dressing percentage (%) = (carcass weight / fasting live weight)×100; eviscerated percentage (%) = eviscerated weight / fasting live weight)×100; semi-eviscerated percentage (%) = semi-eviscerated weight / fasting live weight×100; breast muscle rate (%) = breast muscle weight / fasting live weight×100; leg muscle rate (%) = leg muscle weight / fasting live weight×100.

### Meat quality and sensory determination

#### Meat quality assessment

Post-slaughter, the pectoral muscles for each group of chickens were assessed using a FoodScan Meat Analyzer (Eo Medical Equipment,) to quantify moisture, total protein, and total intramuscular fat levels. A flesh color meter was calibrated before pectoral muscle samples were obtained. The pectoral muscle was excised under consistent temperature and lighting conditions. Meat color was determined using a color difference meter (NR20XE; 3NH Technology) that was calibrated before the pectoral muscle samples were obtained; readings were taken twice, and the average value was computed.

#### pH measurement

After 60 min of sampling, two cross-shaped incisions were made on the left pectoral muscle using a knife, and the pH at the center of each incision was determined twice using a Testo 205 pH meter (Testo). The mean pH of the 60 min sample was determined.

#### Shear force measurement

A rectangular specimen of the pectoral muscle without tendons, adipose tissue, and muscle fascia was obtained and oriented following the alignment of muscle fibers. The specimen was subsequently placed in a water bath at 80°C for 5 min and removed. After the sample reached room temperature, a tenderness meter (TS100; Bulader) was used to slice the sample parallel to the direction of the muscle fibers at dimensions of 2×1×1 cm. Three cuts were made, values were recorded, and the average values were calculated in Newton (N).

#### Drip loss measurement

A scalpel was used to excise a rectangular specimen from the pectoral muscle sample; the specimen was subsequently weighed and designated as A1. A sterilized wire was inserted through one end of the sample, and the meat sample was suspended in a fresh-keeping bag inflated with gas and secured. The sample was refrigerated at 4°C. Water loss was measured over 24 h, and the resulting weight was recorded as A2. The drip loss rate was calculated as follows: (A1–A2)/A1×100%.

#### Meat sensory assessment

The entire pectoralis muscles of the bones and skin were stripped, and the connective tissue, fat, and surrounding minced meat were removed. The pectoralis muscle was cleaned by soaking in running water, wrapped with aluminum booklet paper, placed in a small ceramic dish, and steamed in a pot of boiling water until the temperature at the center reached 80°C; the cooking time was subsequently recorded. Steamed meat samples were allowed to cool to 26°C for 10 min, then cut into 1×3 cm strips, placed on a small ceramic plate, numbered, and randomly distributed to two evaluators who were given two unsalted cookies and one cup of warm water. The evaluators followed the instructions to taste and eat the cookies, and they rinsed their mouths with warm water to remove residual taste that may interfere with the next sample. Similarly, they rated the flavor, juiciness, and chewability of the meat and provided sensory information. The evaluators scored the samples using a 5-point scale: 1 = worst, 2 = bad, 3 = acceptable, 4 = good, and 5 = excellent. Each sample rating was the average scores of the two evaluators.

### Determination of antioxidant indicators

Using commercially available biochemistry kits, we determined the activities of superoxide dismutase (SOD; A001-1), total antioxidant capacity (T-AOC; A015-3), glutathione peroxidase (GSH-Px; A005-1), catalase (CAT; A007-1), and malondialdehyde (MDA; A003-1) in the serum and jejunum following the manufacturer’s instructions (Nanjing Jiancheng Bioengineering Institute).

### Determination of intestinal histomorphology and permeability

After dehydration in a graded ethanol sequence, the jejunal specimens were cleaned with xylene and embedded in paraffin. The materials were sliced into 5-μm portions and stained with hematoxylin and eosin. Using an optical microscope (NIKON Eclipse Ci; Nikon Precision), ten randomly selected fields were examined to assess villus height and crypt depth and calculate villus height-to-crypt depth ratios.

The intestinal permeability-related gene expression in the jejunum was quantified using quantitative real-time PCR. Briefly, the jejunum was ground to a powder in liquid nitrogen, and 1 mL of the TRIzol reagent (MD020; Magen) was added. RNA was extracted using precooled chloroform. The supernatant was transferred to a new centrifuge tube, and isopropanol was added to precipitate the RNA. The RNA precipitate was washed twice with 75% ethanol and diluted with diethyl pyrocarbonate-treated water. All operations were performed on ice. RNA quality was determined through agarose gel electrophoresis, and the RNA was quantified using a bio-photometer (NanoDrop2000; Thermo Fisher Scientific). In addition, 1,000 ng of RNA, 5 mmol/L MgCl_2_, 1 μL of RT buffer, 1 mmol/L dNTP, 2.5 U of AMV, 0.7 nmol/L oligodeoxynucleotides d(T), and 10 U of ribonuclease inhibitor (TaKaRa) were used in the reverse transcription reactions (10 μL). Real-time PCR was performed using a *Bio*-*Rad CFXConnect* system (Bio-Rad). Each real-time PCR product was used as a template in a 20 μL PCR containing 0.2 μmol/L of each primer and SYBR Green premix (Accurate Biology); primer sequences are listed in Supplement 2. Primers were designed for exon-intron ligation using the National Center for Biotechnology Information. The PCR cycle began with a 3-min denaturation at 95°C, followed by 40 cycles: 10 s at 95°C, 30 s at 60°C for *Occludin-1*, *Claudin-1*, *ZO-1*, *Mucin-2*, and *GAPDH*, and 30 s at 72°C. A standard curve was plotted to calculate the efficiency of the real-time PCR primers. GAPDH was used as the housekeeping gene. mRNA expression was quantified by comparing CT methods (2^−ΔΔCT^), which was calculated using the normalization method. Amplified product specificity was analyzed using a melting curve. The primer sequences are presented in Supplement 2.

### Determination of welfare indicators

On the last day of experiment, 2 chickens were randomly picked out from each treatment group and assessed for leg strength based on a four-point gait scoring scale (1 = worst, 2 = bad, 3 = acceptable, and 4 = best), as previously described [14].

Feather coverage scores were derived from the study of Heerkens et al [15]. The body was segmented into five components, namely the head, neck, back, tail, and anus. Each component was evaluated on the aforementioned 4-point scale. Every component was categorized on this 4-point scale. Feather scores for each bird was the sum of feather types 1–5 from a minimum score of 5 to maximum possible (best) ranking value at 20.

The daily behaviors of 150 d-old Jiuyuan Black chickens were video recorded and observed. One chicken was randomly selected from each replicate for observation. The recorded behaviors included feeding, drinking, standing, walking, resting, dustbathing, preening, and aggression. Observations were conducted over three consecutive days, starting one hour after feeding at 9:00 a.m. and lasting for 60 min. Behavioral indicators were analyzed by calculating the proportion of time spent on each behavior relative to the total observation period.

### Data analysis

Results are presented as the mean±standard error of mean (SEM). Statistical analysis and chart mapping were performed using the GraphPad software (GraphPad Prism 7.0). A two-tailed Student’s t-test was performed to compare both groups. Statistical significance was defined as p<0.05.

## RESULTS

### Growth performance parameters

**Figure 1:**
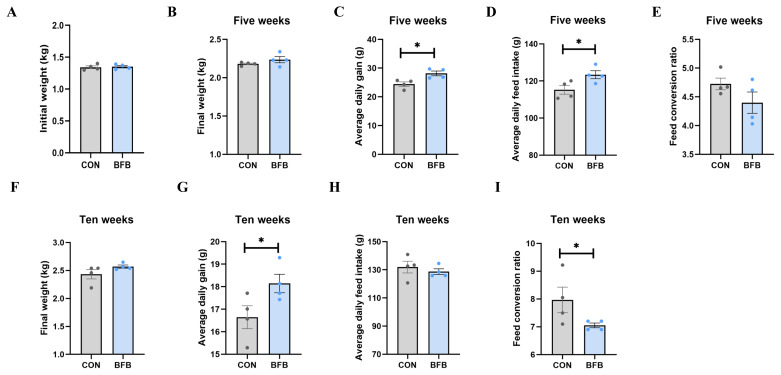
Effects of *Bacillus coagulans* fermentation bedding (BFB) on the growth performance of Jiuyuan Black chickens. Growth performance parameters were evaluated at two time points: the first phase (1–5 wk) and the second phase (6–10 wk) of the experimental period. (A) Initial body weight. (B–E) Growth performance indicators during the first phase. (F–I) Growth performance indicators during the second phase. (B, F) Final body weight at 5 wk and 10 wk, respectively. (C, G) Average daily gain. (D, H) Average daily feed intake. (E, I) Feed conversion ratio. CON: Conventional bedding. Data are shown as the mean±SEM (n = 4, per n including 15 chickens). * p<0.05. SEM, standard error of mean.

As shown in Figure 1 and Supplement 1, the growth parameters of Jiuyuan black chickens varied between the CON and BFB groups. On day 35 of the experiment, chickens in the BFB group showed a significantly higher ADG and ADFI compared to the CON group (p<0.05). By day 70, birds in the BFB group maintained a significantly higher ADG and exhibited a lower FCR than those in the CON group (p<0.05).

### Slaughtering performance parameters

**Figure 2:**
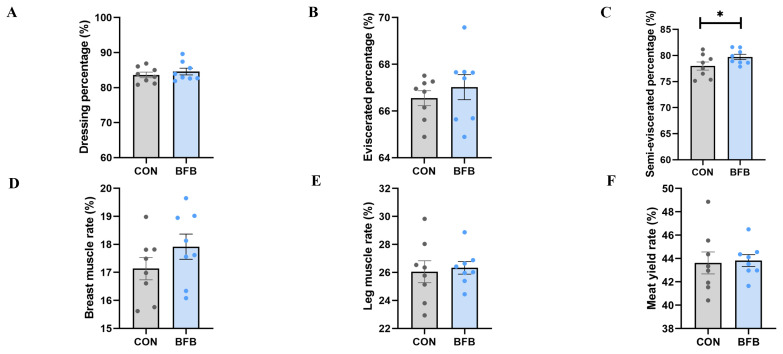
Effects of *Bacillus coagulans* fermentation bedding (BFB) on the slaughtering performanceof Jiuyuan Black chickens during a 10-week experimental period. CON: Conventional bedding. (A) Dressing percentage. (B) Eviscerated percentage. (C) Semi-eviscerated percentage. (D) Breast muscle rate. (E) Leg muscle rate. (F) Meat yield rate. Data are shown as the mean±SEM (n = 8). * p<0.05. SEM, standard error of mean.

Figure 2 shows the slaughter performance parameters of Jiuyuan black chickens in both CON and BFB groups. The semi-eviscerated percentage was significantly higher in chickens from the BFB group than in those in the CON group (p<0.05). However, no significant differences were observed in evisceration percentage, dressing percentage, breast muscle rate, or leg muscle rate between the two groups (p<0.05).

### Organ indices

**Figure 3:**
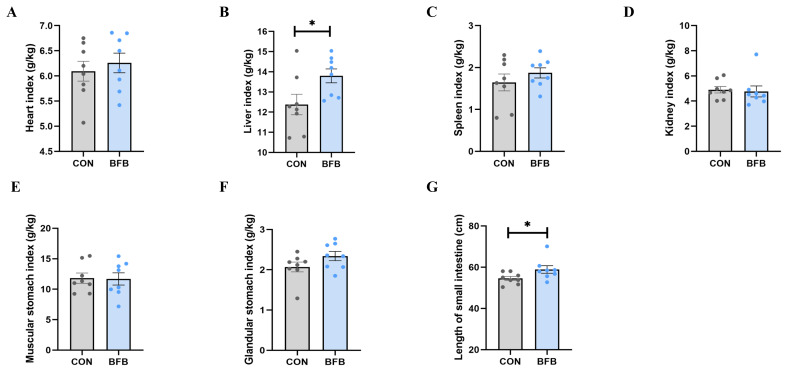
Effects of *Bacillus coagulans* fermentation bedding (BFB_ on the organ indexes of Jiuyuan Black chickens during a 10-week experimental period. CON: Conventional bedding. (A) Heart index. (B) Liver index. (C) Spleen index. (D) Kidney index. (E) Muscle stomach index. (F) Glandular stomach index. (G) Length of small intestine. Data are shown as the mean±SEM (n = 8). * p<0.05. SEM, standard error of mean.

As shown in Figure 3, chickens raised on BFB exhibited a significantly higher liver index and longer small intestine length compared to those in the CON group (p<0.05).

### Meat quality indicators

**Figure 4:**
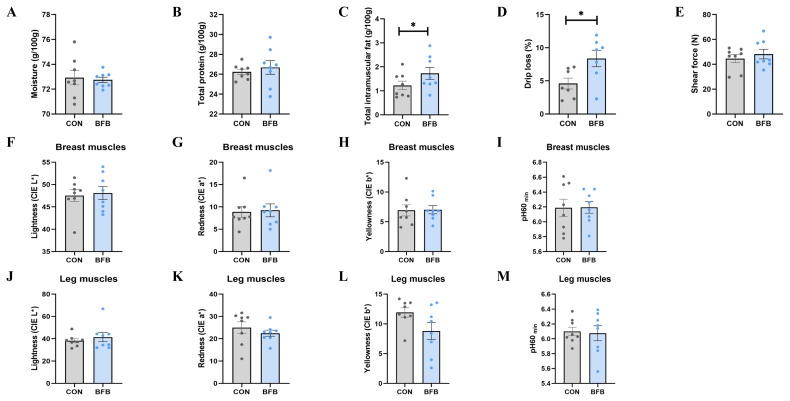
Effects of *Bacillus coagulans* fermentation bedding (BFB) on the meat quality of Jiuyuan Black chickens during a 10-week experimental period. CON: Conventional bedding. (A) Moisture content. (B) Total protein content. (C) Total intramuscular fat content. (D) Drip loss. (E) Shear force. (F–H) Breast muscle color parameters: lightness, redness, and yellowness. (I) Breast muscle pH value. (J–L) Leg muscle color parameters: lightness, redness, and yellowness. (M) Leg muscle pH value. Data are shown as the mean±SEM (n = 8). * p<0.05. SEM, standard error of mean.

**Figure 5:**
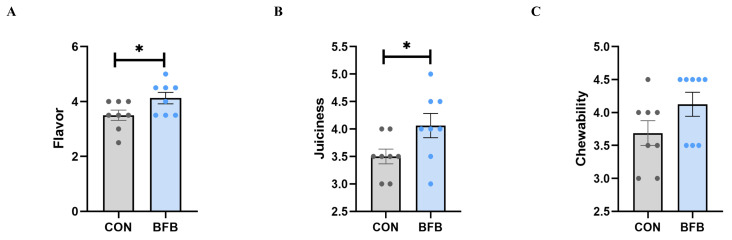
Effects of *Bacillus coagulans* fermentation bedding (BFB) on the breast muscles sensory quality of Jiuyuan Black chickens during a 10-week experimental period. CON: Conventional bedding. (A) Flavor score. (B) Juiciness score. (C) Chewability score. Data are shown as the mean±SEM (n = 8). * p<0.05. SEM, standard error of mean.

The meat quality indicators of the two groups are shown in Figure 4. Total intramuscular fat content and drip loss rate were higher in the breast muscles of chickens in the BFB group compared to those in the CON group (p<0.05). However, no significant differences were observed in meat color between the two groups (p<0.05). Figure 5 shows the meat sensory assessments of the two groups, which shows that chickens in the BFB group exhibited improved flavor and juiciness in breast muscle compared to those in the CON group (p<0.05).

### Intestinal histomorphology and permeability-related genes

**Figure 6:**
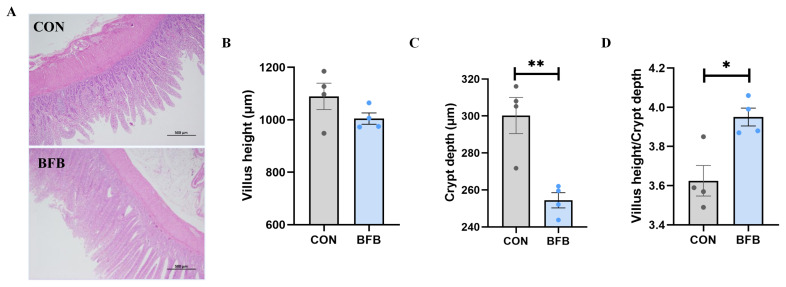
Effects of *Bacillus coagulans* fermentation bedding (BFB) on the intestinal histomorphology of jejunum by magnification 40× in the Jiuyuan Black chickens during a 10-week experimental period. CON: Conventional bedding. (A) Representative images of H&E staining of jejunum. (B) The villus height. (C) The crypt depth. (D) The ratio of villus height and crypt depth. Data are shown as the mean±SEM (n = 4). * p<0.05 and ** p<0.01. SEM, standard error of mean.

**Figure 7:**
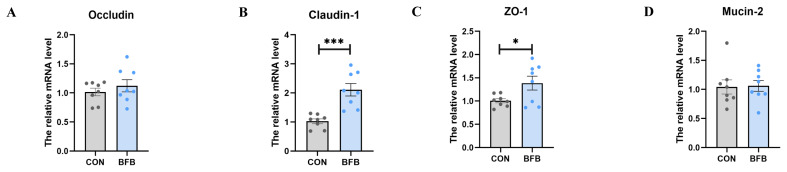
Effects of *Bacillus coagulans* fermentation bedding (BFB) on the intestinal permeability of jejunum in Jiuyuan Black chickens during a 10-week experimental period. CON: Conventional bedding. (A) Occludin. (B) Claudin-1. (C) Zonula occludens-1 (ZO-1). (D) Mucin-2. Data are shown as the mean±SEM (n = 8). * p<0.05 and *** p<0.001. SEM, standard error of mean.

Figure 6 provides a histological analysis of the jejunum, with Figure 6A showing representative images of intestinal morphology. Chickens in the BFB group exhibited significantly lower crypt depth and a higher villus height-to-crypt depth ratio in the jejunum compared to the CON group (p< 0.05), which indicates enhanced nutrient absorption capacity. As illustrated in Figure 7, the relative mRNA expression of *Claudin-1* and *ZO-1* in the jejunum was significantly upregulated in the BFB group compared to the CON group (p<0.05). This suggests improved intestinal barrier integrity in chickens provided with fermented bedding.

### Antioxidant indicators

**Figure 8:**
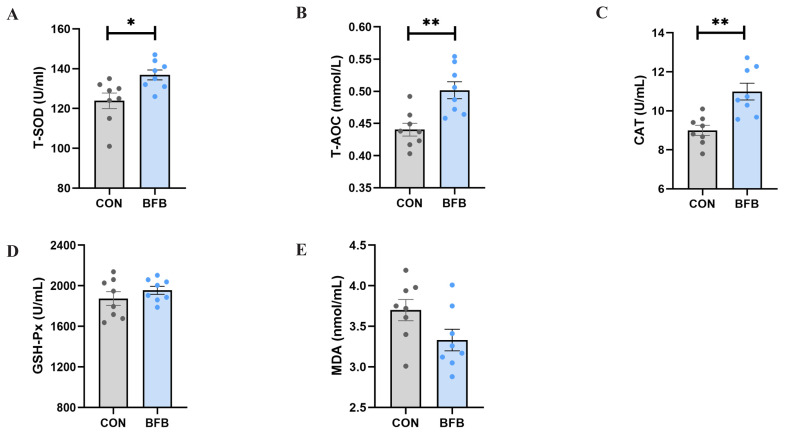
Effects of *Bacillus coagulans* fermentation bedding (BFB) on the antioxidant indexes of serum in Jiuyuan Black chickens during a 10-week experimental period. CON: Conventional bedding. (A) Total superoxide dismutase (T-SOD). (B) Total antioxidant capacity (T-AOC). (C) Catalase (CAT). (D) Glutathione peroxidase (GSH-Px). (E) Malondialdehyde (MDA). Data are shown as the mean±SEM (n = 8). * p<0.05 and ** p<0.01. SEM, standard error of mean.

**Figure 9:**
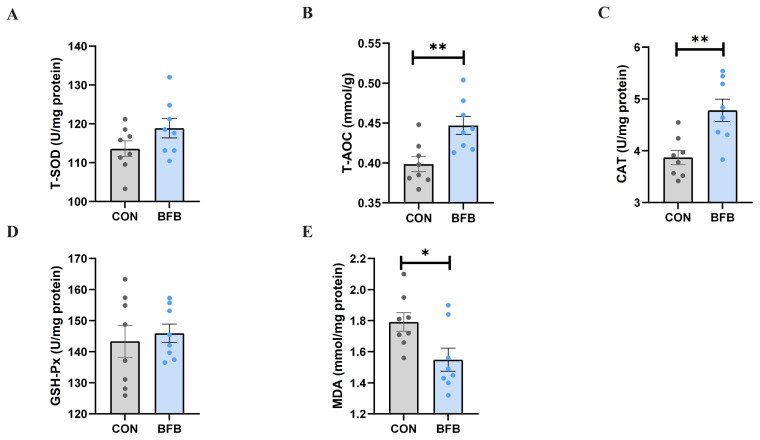
Effects of *Bacillus coagulans* fermentation bedding (BFB) on the antioxidant indexes of jejunum in Jiuyuan Black chickens during a 10-week experimental period. CON: Conventional bedding. (A) Total superoxide dismutase (T-SOD). (B) Total antioxidant capacity (T-AOC). (C) Catalase (CAT). (D) Glutathione peroxidase (GSH-Px). (E) Malondialdehyde (MDA). Data are shown as the mean±SEM (n = 8). * p<0.05 and ** p<0.01. SEM, standard error of mean.

Figures 8, 9 show the antioxidant indices of the Jiuyuan black chickens in the serum and jejunum of the two groups, respectively. Chickens in the BFB group exhibited increased T-AOC levels and CAT activity in both serum and jejunum than to those in the CON group (p<0.05). Furthermore, T-SOD activity was significantly higher in the serum, whereas MDA content was significantly lower in the jejunum of chickens in the BFB group (p<0.05). These findings suggest enhanced oxidative stress resistance**.**

### Overall welfare of the chickens

**Figure 10:**
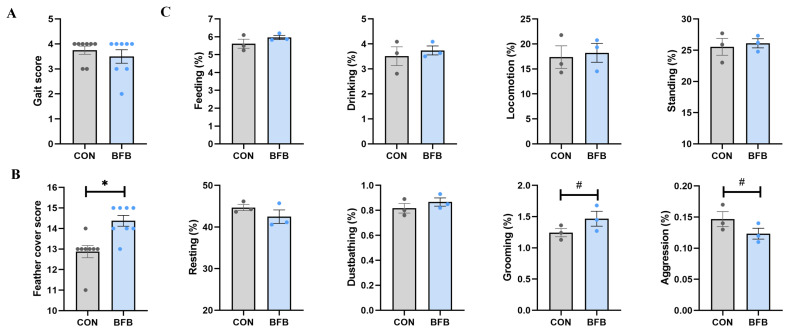
Effects of *Bacillus coagulans* fermentation bedding (BFB) on the welfare indicators of Jiuyuan Black chickens during a 10-week experimental period. CON: Conventional bedding. (A) Gait score (n = 8). (B) Feather cover score (n = 8). (C) Behavioral indicators (n = 3). Data are shown as the mean±SEM. 0.05<^#^ p< 0.1 and * p<0.05. SEM, standard error of mean.

Figure 10 illustrates the welfare indicators of the birds in both groups. Chickens in the BFB group exhibited a significantly higher feather cover score compared with those in the CON group (p<0.05). Furthermore, the percentage of grooming behavior had an increased tendency, while aggressive behavior had a decreased tendency in the BFB group (0.05<p<0.1), which indicates improved welfare conditions in chickens raised on BFB.

## DISCUSSION

ADG is a key determinant of feed efficiency, directly influencing growth rates and feed expenditure, while FCR serves as an indicator of economic viability in poultry farming [4,16]. Throughout the 10-week study period, chickens reared on BFB exhibited a higher ADG and lower FCR than those reared using CON, indicating substantial improvements in overall growth performance and feed utilization efficiency. Further carcass trait analysis revealed that chickens in the BFB group had a significantly high semi-eviscerated yield percentage, indicating an increased proportion of edible meat per unit of body weight [17]. This phenomenon may be attributed to the digestive-enhancing properties of *B. coagulans*, particularly its ability to convert fecal matter into energy and fertilizer [18]. Additionally, we observed an increase in preening behavior in the BFB group, suggesting that chickens might ingest active *B. coagulans* spores and their beneficial metabolites from the litter through preening in a continuous, low-dose manner, which could enhance feed utilization efficiency.

The breast muscles of chickens reared on BFB showed increased intramuscular fat and drip loss. This finding is consistent with that of an earlier study indicating a positive relationship between intramuscular fat content and drip loss [11]. Since intramuscular fat contributes to flavor profile and overall meat quality [19], maintaining optimal levels is crucial for consumer preference and market value [20]. Furthermore, at the end of the trial, BFB increased the flavor and juiciness of chicken breast muscle, indicating an increase in meat sensory quality [21]. Fat deposition may contribute to enhancing meat flavor and juiciness, further contributing to improving the overall sensory quality [22]. Therefore, chickens in the BFB group outperformed those in the CON group in terms of meat quality.

The intestinal morphology is a key indicator of nutrient absorption and overall gut health [23,24]. Chickens grown on BFB treatment exhibited a reduced jejunal crypt depth and increased villus height-to-crypt depth ratio, indicating a healthy, mature intestinal lining and suggesting enhanced nutrient absorption and digestion [25]. Moreover, BFB treatment also significantly increased the expression of tight junction proteins *Claudin-1* and *ZO-1* in the jejunum. These proteins are essential for the intestinal barrier, and their increased expression indicates an enhanced mucosal defense, potentially limiting the translocation of luminal pathogens and antigens into the systemic circulation [26]. Enhancing intestinal barrier function with BFB can lower the metabolic demands of systemic immune activation, allowing more energy and nutrients to be directed toward growth and weight gain [27], as shown by increased ADG and semi-eviscerated yield percentage. Furthermore, a significant increase in the length of the small intestine was observed for Jiuyuan black chickens treated with BFB. A longer small intestine increases the surface area for nutrient absorption [28]. This is likely a response of the digestive system to long-term adaptation in BFB treatment; it adjusts progressively for better use of nutrients given with BFB treatment.

The antioxidant defense system is an essential part of the cellular protection from oxidative stress and damage in animals [29]. Chickens raised on BFB exhibited higher T-SOD and CAT activities and higher T-AOC levels. Oxidative stress is characterized by an imbalance between the generation and elimination of reactive oxygen species (ROS) [30]. In livestock and poultry production, systemic low-grade inflammation serves as a major contributor to ROS generation, a condition frequently originating from intestinal dysfunction [31]. Our results suggest that BFB treatment markedly improves intestinal barrier integrity. A well-functioning intestinal barrier plays a crucial role in preventing the translocation of pro-inflammatory agents, such as bacterial endotoxins, into the portal venous system [32,33]. We speculated that BFB may mitigate the translocation of enteric endotoxins into the circulatory system by improving the intestinal barrier, thereby attenuating endotoxin-induced immune-inflammatory responses and the consequent excessive production of ROS. As a result, the observed concurrent increase in SOD and CAT activities in the BFB-treated group suggests an adaptive defensive response by the organism to effectively neutralize superoxide anions and inhibit the formation of hydroxyl radicals [34,35]. Conversely, chickens in the BFB-treated group showed low levels of MDA, indicating reduced lipid peroxidation within the cells [36]. Lipid peroxidation can disrupt cell membrane integrity, affect membrane fluidity, and impair the function of membrane-bound proteins and receptors, all of which can have far-reaching consequences for cellular homeostasis [37]. The overall enhancement in T-AOC signifies that BFB enhances the oxidative stability of Jiuyuan black chickens.

Feather coverage is a crucial indicator of welfare for poultry animals, as feathers can protect chickens from environmental stressors, help maintain body temperature and reduce thermal energy loss [38,39]. In this study, chickens reared on exhibited improved feather integrity, suggesting enhanced physical welfare. Furthermore, chickens in the BFB group displayed reduced aggressive behavior and increased grooming tendencies. We posited that the cessation of continuous energy expenditure on stress responses allows for the reallocation of energy towards routine, tranquil behaviors, such as preening, and metabolic processes essential for maintaining high-quality plumage, likely resulting in enhanced physiological and behavioral welfare [40]. For broiler chickens, fermented bedding promoted feather coverage and had beneficial effects on the behavior of birds that had a multifaceted positive welfare outcome.

Overall, BFB treatment optimized the structure of the intestine and enhanced intestinal barrier function, thus increasing antioxidant levels, and leading to a positive influence on growth performance and meat quality in Jiuyuan Black chickens.

## CONCLUSION

In this study, we have confirmed that adding BFB to Jiuyuan Black chicken breeding can promote growth performance and improve meat quality, optimize intestinal structure as well as strengthen antioxidant defense. BFB also had a positive impact on poultry welfare, as it improved feather scores and decreased aggressive behaviors. This study confirms that BFB can be used as an alternative bedding material for sustainable poultry production which not only helps in maintaining the economic viability but also provide environmentally friendly solutions to litter disposal. Further studies are needed to elucidate the synergistic effects of this additive with other eco-feed additives that can ensure continued efficacy through successive periods of production.

## Figures and Tables

**Figure f11-ab-250646:**
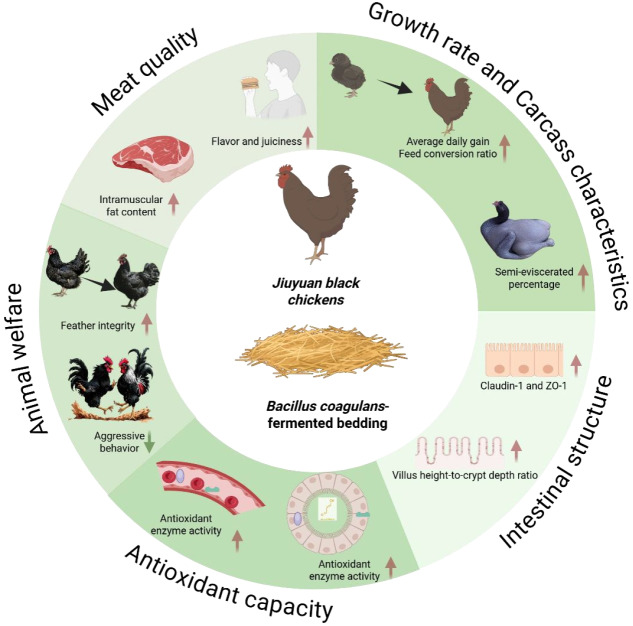


## Data Availability

Upon reasonable request, the datasets of this study can be available from the corresponding author.

## SUPPLEMENTARY MATERIAL

**Supplement 1.** Ingredients and nutrient composition of the experimental diet (as-fed basis, %).

**Supplement 2.** Primers used for real-time fluorescent quantitative PCR analysis.
