# Supplementary material for: Enhancing growth performance, meat quality, and gut health of Jiuyuan Black chickens by using Bacillus coagulans-fermented bedding
Source: Anim Biosci. 2025 Dec 1;39(6):250646. doi: 10.5713/ab.250646 (PMC13243926; doi:10.5713/ab.250646)
Supplement: Supplementary file 1 [file ab-250646-Supplementary-1.pdf]

Supplement 1. Ingredients and nutrient composition of the experimental diet (as-fed basis, %)

**Items**	**Finisher (days 80–150)**
Ingredients (%)	
Corn	70.5
Soybean meal	14.0
Fish meal	3.00
Wheat	4.50
Rice bran	4.00
Soybean oil	1.00
Calcium hydrogen phosphate	0.80
Calcium carbonate	1.00
Salt	0.20
Premix[1]	1.00
Nutrient composition [2](%)	
Crude protein (CP)	15.0
Calcium (Ca)	0.76
Non-phytic acid (NPP)	0.35
Lysine	0.71
Methionine + Cystine	0.48
ME[b] (kcal/kg)	2940

[1)] Composition per kg of premix: vitamin A 10000 IU/kg, vitamin D_3 3000 IU/kg, vitamin E 20 IU/kg, vitamin K 32 mg/kg, thiamine 1 mg/kg, riboflavin 8 mg/kg, calcium pantothenate 40 mg/kg, niacin 32.5 mg/kg, pyridoxine 8 mg/kg, biotin 0.2 mg/kg, folic acid 1.5 mg/kg, vitamin B_{12} 0.05 mg/kg, choline 500 mg/kg, manganese 70 mg/kg, Iodine 1 mg/kg, iron 80 mg/kg, copper 8 mg/kg, zinc 80 mg/kg.
[2)] Calculated values according to the data of Chinese Table of Feed Ingredients and Nutritional. Abbreviation: ME, metabolizable energy.
