# Supplementary material for: Enhancing growth performance, meat quality, and gut health of Jiuyuan Black chickens by using Bacillus coagulans-fermented bedding
Source: Anim Biosci. 2025 Dec 1;39(6):250646. doi: 10.5713/ab.250646 (PMC13243926; doi:10.5713/ab.250646)
Supplement: Supplementary file 2 [file ab-250646-Supplementary-2.pdf]

Supplement 2. Primers used for real-time fluorescent quantitative PCR analysis

**Gene**	**Sequences (5' to 3')**	**Annealing temperature (°C)**	**Accession No.**
GAPDH	F: TCCTCCACCTTTGATGCG<br>R: GTGCCTGGCTCACTCCTT	60	NM_204305.2
Occludin-1	F: TCGACAGCATCACCGAGGACA<br>R: CTCTCCTGCTTCTTGCTTTGGTA	60	NM_205128.1
Claudin-1	F: CCACGTCATGGTATGGCAAC<br>R: AAGAGGGCTGATCCAAACTCA	59.5	NM_001013611.2
Zonula Occludens-1	F: ACGAGCTACACTGTTGAATGTCC<br>R: ATGATCTGATGCCAGCGACT	59.5	XM_015278975.1
Mucin-2	F: GTGCCCATCTCCAAATGCC<br>R: TGTCACTGAAGTACACCGGAT	59.5	XM_040701656.2
